# Dilated Cardiomyopathy and Aortic Thrombosis in a Capuchin Monkey ( *Sapajus apella* )

**DOI:** 10.1111/jmp.70079

**Published:** 2026-05-11

**Authors:** Christiane Helm, Andreas Bracke, Jens Peter Teifke, Reiner Ulrich

**Affiliations:** ^1^ Institute of Veterinary Pathology, Faculty of Veterinary Medicine Leipzig University Leipzig Germany; ^2^ District of Anhalt‐Bitterfeld Consumer/Veterinary Protection and Health Department Köthen Anhalt Germany; ^3^ Veterinary Practice Quandt & Bracke Greifswald Germany; ^4^ Department of Experimental Animal Facilities and Biorisk Management Friedrich‐Loeffler‐Institute Greifswald‐Insel Riems Germany

**Keywords:** cardiomyopathy, mural aneurysm, myocardial fibrosis, primate, thromboembolism

## Abstract

This case report describes a capuchin monkey ( *Sapajus apella* ) that presented with bilateral paralysis and cold hind limbs. Clinical examination, including ultrasound imaging, revealed dilated cardiomyopathy and aortic thrombosis. Macroscopic, histopathological, and immunohistochemical analyses confirmed these findings. The structural myocardial changes are comparable to those of human cardiomyopathy.

## 1 Introduction

Cardiac diseases are a common cause of morbidity and mortality in primates, including humans [1]. Cardiomyopathies are characterized by structural and functional abnormalities of the myocardium and are classified as primary, secondary, or mixed forms [2, 3, 4, 5]. Dilated cardiomyopathy (DCM) is associated with ventricular dilatation and systolic dysfunction and may lead to congestive heart failure or sudden death [6].

The composition of the extracellular matrix (ECM) influences the functional capacity of the myocardium, as it consists of a complex network of structural proteins, including 85% collagen type I, 15% collagen type III, and up to 5% collagen type V [7, 8]. In DCM, the ECM commonly displays increased amounts of collagen types I and III [7, 9]. Fibrotic remodeling is driven by fibroblast‐derived matrix metalloproteinases (MMP), including MMP‐1 and profibrotic cytokines such as TGF‐β1 and TGF‐β2, which activate collagen gene expression [7, 10].

This case report aims to describe the clinical signs, macroscopic lesions, and histopathologic findings in a case of DCM in a capuchin monkey, with a focus on the major regulators and structural components of fibrosis.

## 2 Case Report

A 20‐year‐old male capuchin monkey ( *Sapajus apella* ), weighing 4 kg, was housed in a municipal zoological park in Grimmen, Germany, together with another male and one female under standard conditions with access to indoor and outdoor enclosures. The animal presented with bilateral paralysis and cold hind limbs. Clinical examination revealed absent femoral pulses and no interphalangeal reflex. Heart auscultation showed a holosystolic murmur (2/6) over the mitral valve and a doubled second heart sound. Lung auscultation was unremarkable.

Radiography ruled out fractures and intervertebral disc disease. Electrocardiography revealed sinus rhythm (120 bpm) with flat T waves and isoelectric ST segments. Echocardiography demonstrated a hypokinetic, dilated left ventricle with thinning of the posterior wall (mural aneurysm), moderate mitral insufficiency, and an occluding thrombus in the abdominal aorta.

**FIGURE 1:**
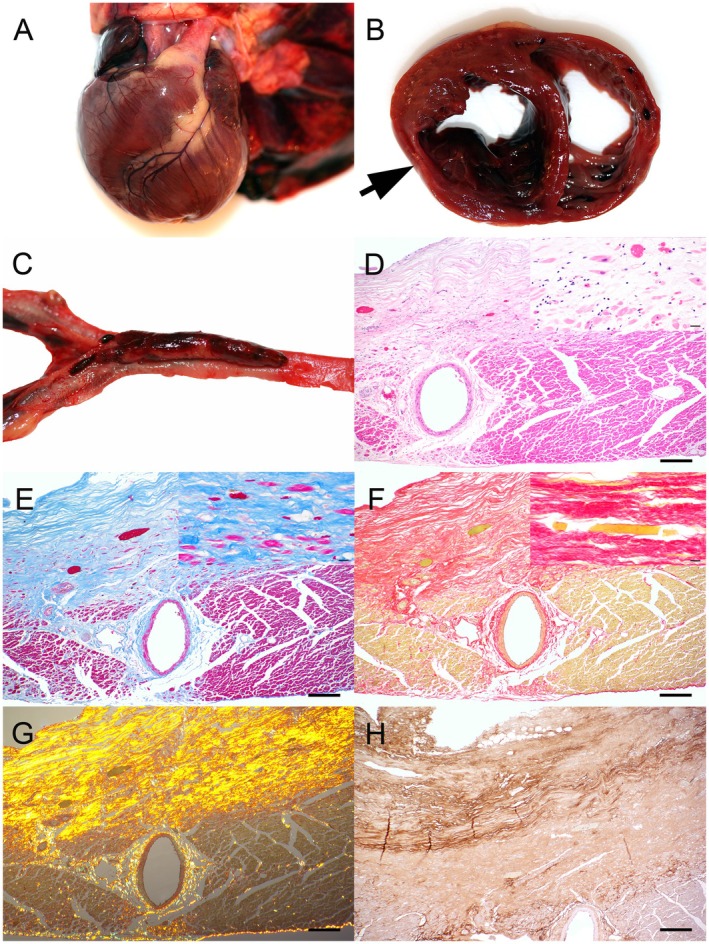
Gross, histopathological, and immunohistochemical findings in the myocardium. (A) Gross appearance of the heart showing a globular shape characteristic of dilated cardiomyopathy. (B) Left ventricular wall with localized reddish‐gray thinning (arrow) corresponding to the mural aneurysm detected by ultrasound. (C) Adherent thrombus in the abdominal aorta at the iliac bifurcation. (D) Low‐power view of myocardial degeneration and interstitial fibrosis in the left ventricular wall (H&E, scale bar = 200 μm); inset: Higher magnification showing cardiomyocyte shrinkage with pyknotic nuclei, myofiber loss, fibrosis, and lymphohistiocytic infiltration (scale bar = 20 μm). (E) Azan stain highlighting interstitial collagen deposition (scale bar = 200 μm); inset: Higher magnification showing marked loss of cardiomycytes with extensive fibrosis (scale bar = 20 μm). (F) Picrosirius red stain highlighting interstitial collagen deposition (scale bar = 200 μm); inset: Higher magnification showing cardiomyocyte loss with surrounding collagen‐rich fibrosis (scale bar = 20 μm). (G) Picrosirius red under polarized light showing birefringent collagen fibers (scale bar = 200 μm). (H) Immunohistochemistry for collagen type III showing strong extracellular expression in fibrotic regions (scale bar = 200 μm).

Due to the poor prognosis, the animal was euthanized by intravenous administration of 7 mL of pentobarbital (500 mg/mL). A complete necropsy was performed according to standard procedures under biosafety level 2 conditions. The heart weighed 28 g (0.7% of body weight) and exhibited a spherical shape due to left ventricular dilatation (Figure 1A). The mural aneurysm area appeared grayish‐red and thinned (Figure 1B). A 4.5 cm adherent thrombus was present in the abdominal aorta at the iliac bifurcation (Figure 1C). Additional findings included a gallbladder stone, renal cysts, a unilateral Leydig cell tumor, pulmonary congestion, edema, and splenic congestion.

Tissue samples of skeletal musculature, heart, aorta, lungs, spleen, liver, gallbladder, jejunum, colon, kidneys, testes, and brain were fixed in 4% neutral buffered formaldehyde, processed, and embedded in paraffin wax. Sections (2–3 μm) were stained with hematoxylin and eosin (H&E), Azan and picrosirius red. Histopathology revealed severe chronic myocardial degeneration with cardiomyocyte atrophy, loss of myofibers, extensive fibrosis, and mild to moderate lymphohistiocytic infiltration (Figure 1D,E). The most affected areas corresponded to the macroscopically thinned region with near‐complete cardiomyocyte loss replaced by dense collagen. Fibrotic changes extended into the papillary muscles, septum, and atrioventricular regions. Picrosirius red confirmed an increased presence of red collagen fibers exhibiting yellow‐orange birefringence under polarized light (Figure 1F,G).

Immunohistochemistry using the avidin‐biotin‐peroxidase complex (ABC) method with 3,3′‐diaminobenzidine as the chromogen and hematoxylin counterstain showed increased expression of type III collagen (Figure 1H) and TGF‐β1, whereas MMP‐1, collagen VI, and TGF‐β2 were reduced (Table 1).

**TABLE 1:** Antibodies used for immunohistochemistry and immunoreactivity observed in the scarred and normal‐appearing myocardium in the present case.

Antigen (primary antibody)	Producer	Clonality	Dilution, pretreatment	Function	Test results
Matrix metalloprotease‐1 (MMP‐1)	LAB VISION	Polyclonal, rabbit	1:25	Cleaves collagen types I, II, III, VII, VIII and X	30% of the scar tissue positive, 70%–80% of the unchanged myocardium positive
	#RB‐1536‐P0		Protease		
Collagen III	Acris, CL197P	Polyclonal, rabbit	1:1000	Mainly granulation tissue, skin and perivascular tissue	40% of scar tissue positive
			None		20%–30% of unchanged myocardium (endomysium) positive
Collagen VI	Quartett, #031500605	Polyclonal, rabbit	1:100	Mainly interstitial connective tissue	75% of scar tissue positive, 90%–100% of unchanged myocardium (endomysium) positive
			Protease		
Transforming growth factor‐*β*‐1 (TGF‐*β*‐1)	Acris	Monoclonal, mouse	1:1500	Regulation of proliferation, differentiation and apoptosis	30% of the scar tissue positive, 10%–20% of the unchanged myocardium positive
	DM1047	Clone TB21	Citrate buffer		
Transforming growth factor‐*β*‐2 (TGF‐*β*‐2)	Santa Cruz	Polyclonal, rabbit	1:50	Regulation of proliferation, differentiation and apoptosis	40%–50% of the scar tissue positive, 70%–80% of the unchanged myocardium positive
	sc‐90		None		

## 3 Discussion

This case report presents the clinical and postmortem findings in a capuchin monkey with DCM and aortic thrombosis. DCM has previously been reported in owl monkeys (*Aotus* sp.) [11, 12], spider monkeys (*Ateles* sp.) [13, 14], tamarins (*Sanguinus mystax*) [15], woolly monkeys ( *Lagothrix lagotricha* ) [16], a marmoset ( *Callithrix penicillata* ) [17], a De Brazza's monkey ( *Cercopithecus neglectus* ) [18], a chimpanzee ( *Pan troglodytes* ) [19], and a squirrel monkey ( *Saimiri sciureus* ) [20].

The myocardial lesions, characterized by cardiomyocyte atrophy, fibrosis, and lymphohistiocytic infiltration, are considered idiopathic; however, a subclinical or past infection cannot be excluded. The etiologic differential diagnoses for DCM include genetic mutations, nutritional deficiencies, toxins, infectious agents, and endocrine or autoimmune disorders [14, 18, 20, 21, 22]. Hypertension [16], and stress from captivity have also been implicated [11].

The absence of signs of chronic congestion in the lungs or liver suggests that the DCM was clinically compensated until the formation of the thrombus. Aortic thromboembolism is well recognized in feline cardiomyopathy [23]. In the present case, occlusion of the abdominal aorta resulted in acute hind limb paralysis. Comparable cases of thromboembolism‐associated paralysis have been described in tamarins [24] and owl monkeys [25]. Intracardiac or aortic thrombus formation has been reported previously in capuchin monkeys, in association with an experimental infection with herpes simplex virus type 2 [26]. A woolly monkey presented with intracardial thrombus formation in association with coagulase‐positive *Staphylococcus* spp. [27].

Paraplegia is a form of bilateral hind limb paralysis characterized by motor dysfunction, sensory deficits, and autonomic impairment [28, 29, 30]. In addition to vascular causes such as aortic thrombosis, differential diagnoses for paraplegia in nonhuman primates include traumatic spinal cord injury [31, 32] and nontraumatic causes such as fibrocartilaginous embolism [33], congenital malformations, neoplasia [34], or infectious diseases [35, 36].

The circumscribed bulge of the left ventricular wall with a thin fibrotic wall is termed “mural aneurysm” by cardiologists [37]. Picrosirius red confirmed fibrosis. Under polarized light, an orange‐red color indicates densely packed, mature collagen fibers, whereas a yellow‐green color indicates loose, immature collagen fibers [38]. In this case, structural remodeling mirrors findings in human and canine DCM, including increased collagen I and III, reduced MMP‐1 and elevated TGF‐β1 [6, 7, 10]. Type I collagen increases stiffness and impairs function, while type III collagen enhances elasticity [7, 39].

Although therapeutic options for DCM exist, their efficacy remains limited [40], and the overall prognosis is generally poor [18]. A better understanding of myocardial fibrosis and extracellular matrix dysregulation may contribute to the development of future therapeutic approaches [8, 21].

## Funding

The publication of this article in the open‐access format was supported by the Open Access Publishing Fund of Leipzig University.

## Ethics Statement

The authors confirm that the ethical policies of the journal, as noted on the journal's author guidelines page, have been adhered to. Ethical approval was not required because no animals were used for research in this study.

## Conflicts of Interest

There is no conflicts of interest. The topic was presented as a poster at the DVG Veterinary Pathology Conference in Fulda, Germany, in 2020.

## Data Availability

The data that support the findings of this study are available from the corresponding author upon reasonable request.
